# Exploring the influence of a user-specific explainable virtual advisor on health behaviour change intentions

**DOI:** 10.1007/s10458-022-09553-x

**Published:** 2022-04-04

**Authors:** Amal Abdulrahman, Deborah Richards, Ayse Aysin Bilgin

**Affiliations:** 1 School of Computing, Macquarie University, Balaclava Road, Sydney, 2109 NSW Australia; 2 School of Mathematical and Physical Sciences, Macquarie University, Balaclava Road, Sydney, 2109 NSW Australia

**Keywords:** Explainable agents, Personal virtual advisor, Reason explanation, Behaviour change intention, Working alliance, Trust

## Abstract

Virtual advisors (VAs) are being utilised almost in every service nowadays from entertainment to healthcare. To increase the user’s trust in these VAs and encourage the users to follow their advice, they should have the capability of explaining their decisions, particularly, when the decision is vital such as health advice. However, the role of an explainable VA in health behaviour change is understudied. There is evidence that people tend to change their intentions towards health behaviour when the persuasion message is linked to their mental state. Thus, this study explores this link by introducing an explainable VA that provides explanation according to the user’s mental state (beliefs and goals) rather than the agent’s mental state as commonly utilised in explainable agents. It further explores the influence of different explanation patterns that refer to beliefs, goals, or beliefs&goals on the user’s behaviour change. An explainable VA was designed to advise undergraduate students how to manage their study-related stress by motivating them to change certain behaviours. With 91 participants, the VA was evaluated and the results revealed that user-specific explanation could significantly encourage behaviour change intentions and build good user-agent relationship. Small differences were found between the three types of explanation patterns.

## Introduction

Intelligent virtual agents (IVAs) have become more acceptable in our world in various fields such as health [1], education [2] and marketing [3]. To increase the acceptability of IVAs, those agents must be transparent by explaining their behaviour. Explainability can increase the users’ comprehension [4], satisfaction [5], and more importantly the human-agent trust [6] which plays a significant role towards achieving the system goals (e.g. behaviour change [7]). How an IVA can communicate/explain the reasoning behind its behaviour is an important open problem for developing believable and acceptable explainable agents (XAs) [8].

The vast body of research in the human-agent interaction for behaviour change field is built on theories and findings from the social sciences. Some of this work has sought to build XAs that mimic the natural human method of explanation. For example, earlier [9], stated that, similar to a human being, an agent can be represented with three stances: physical, design, and intentional stances. While the physical and design stances refer to the hardware and software that construct an artificial agent as an entity, the intentional stance is the rational cognitive representation of the agent which can explain and predict the agent’s current and future intentional actions [10].

[11] distinguished explaining the intentional behaviours from the unintentional behaviours. People use reason explanation by referring to the reason that derived the behaviour to explain intentional behaviours and causal explanation by referring to the actual causes of unintentional behaviours. In reason explanation, people commonly refer to their mental state (i.e. beliefs and desires/goals) as reasons behind their intentional behaviours. For example, a man explained why he is resigning from his job by saying “because I think there is lack of career progression in this company [belief] and I want a higher position than I have now [goal]”.

Inspired by the Belief-Desire-Intention (BDI) model by [12], BDI agents have been introduced which include beliefs and goals besides intentions as the main components to drive the agent’s actions [13, 14]. The design of BDI agents facilitates the implementation of XAs that use reason explanation to explain their intentional behaviours. This ability of BDI agents is important because human users regard the agent’s behaviours as intentional behaviours and they expect to receive a similar explanation from the agent to what they receive from humans when they explain their intentional behaviour [15].

The explanation process is a social process undertaken in a conversational form to close a gap in understanding between the explainer and the explainee [16]. The gap could be the provided knowledge itself or the inference of the provided knowledge [17]. According to the conversational model by [18], an explanation must appropriately answer the *why* question and must be relevant to the explainee. Explanation relevancy could be connected to providing the relevant reasons behind the action (beliefs and/or goals) [19, 20] or the relevant information to the explainee’s context [21]. However, an agent may derive its behaviour as a result of a series of beliefs and goals, and including all of them in the explanation may generate a long and irrelevant explanation [22]. Selecting the relevant knowledge or the elements to build the reason explanation using beliefs and goals is challenging.

Proper explanation improves user-agent understanding and trust and, consequently, increases the user’s intention to follow the advice recommended by the virtual advisor [23]. Motivating a user to change a behaviour is more effective when the motivation is internalised which has been shown to occur when the persuasion attempt is aligned with the user’s cognitive state: beliefs and goals [24]. However, so far, the introduced XVAs refer to their own cognition rather than the user’s cognition which undermines the concept of personalising the delivered message/recommendation to change a behaviour according to the user’s thoughts and reasoning. In general, personalisation in the field of explainable agency is still very limited: about 8% of the current literature [25]. Thus, we distinguish between the agent’s behaviour and the user’s behaviour and believe that explanation should include the user’s beliefs and goals when the recommendations are behaviours required to be performed by the user, not by the agent. Hence, in our study, we investigate the following question: *How do agent’s explanations that refer to the user’s beliefs or goals influence the user’s intention to change the behaviours recommended by the agent?*. We contribute to the body of research to find relevant user-specific explanation patterns that could persuade a user to adopt healthier behaviours.

The theory of reasoned action, a well-known theory in social psychology, posits that the strongest predictor of a person’s actual volitional behaviour is one’s intention towards this behaviour [26]. Thus, we are measuring the behaviour change intentions as an indication of the actual behaviour change.

The rest of the paper is organized as follows: in Sect. 2, we review the most related work to this research that leads to form our research hypotheses. In Sect. 3, we present the cognitive architecture of the proposed XVA followed by how we designed our research and the methods used to test the hypotheses in Sect. 4. Section 5 presents the experimental results followed by a discussion in Sect. 6 and conclusion in Sect. 7.

## Literature review

Non-adherence to heath recommendations is a substantial complex problem [27]. For example, the rate of non-adherence to prescribed medication only in developed countries reached 50% and it is more likely to be higher in the developing countries and this rate increases to 70% when the professional recommendation includes behaviour change [28]. Non-adherence to the recommended actions by a healthcare giver could be unintentional or intentional behaviour. Unintentional non-adherence is usually a result of the patient’s forgetfulness or misunderstanding whereas intentional non-adherence is a result of the patient’s choice not to take the recommended action [29]. Reminders, simplification, and education have been found to be successful interventions to deal with unintentional non-adherence behaviour [30, 31]. However, the intentional non-adherence is a more complex problem and psychological interventions that target the patient’s cognition and emotions are more successful in motivating the patients to sustain a behaviour for a longer time [32].

In health, the therapist-patient relationship, also called working alliance (WA), is regarded as a good predictor of patient adherence [33] and fostering the patient’s behaviour change [7, 34]. The patient’s active participation in treatment planning can boost the WA and consequently increase adherence to the therapist’s recommendations including health behaviour change [35, 36]. Different factors can shape the patient’s engagement in the planning process such as the patient’s personal beliefs, desires, treatment goals, and social status [37].

The use of an IVA as a virtual advisor/therapist is compelling in the health domain to provide advice and/or support [38, 39]. Over the past decade, several IVAs have been developed to improve the lifestyle of users with health problems such as obesity and mental health [40]. IVAs can build positive user-agent relationship via the inclusion of relational and emotional cues [41]. However, there is no firm evidence of the efficiency of these cues in changing the users’ behaviours [42]. Persuading patients/users towards healthier behaviour change is found to be more effective when the agent’s recommendation messages are properly designed and personalised according to users’ preferences [43, 44].

The term persuasion is commonly used to refer to the process of persuading someone (persuadee) to change a belief or a behaviour. Many strategies have been proposed and evaluated in the domain of human-computer interaction such as Cialdini’s strategies [45, 46]. Stated that a system can be a persuasion system if one or more social cues are included in the system such as embodiment, social language, and empathy. For behaviour change [47], argued that persuasion is not normative and any argument can be called a persuasive argument as long as it persuades the user to do/do not change a behaviour. On the other hand [48], argued that to sustain a persuasion attempt to change a user’s intention towards a behaviour, the persuasive message should be built on the basis of the user’s beliefs, values, and goals. This postulation is in line with our persuasion argument (user-specific reason explanation); however, they further postulated that persuasion is a multi-phase process where the user’s beliefs and goals must be evaluated during the interaction to adapt the persuasion process. Hence, in this paper, we use the word persuade as a synonym to motivate and encourage rather than claiming to propose a persuasion system. Further, in our approach there is no intention to persuade a user to change his/her beliefs, for example, as in persuasion theory [49].

### IVAs for health behaviour change

A great number of IVAs have been introduced to address the problem of adherence and behaviour change. Some of these IVAs are designed to build a user-agent relationship through the use of verbal or nonverbal empathy [39, 50, 51]. An example of an empathic agents for health applications is Ellie [52] that was designed with appropriate verbal and non-verbal emotional behaviours to encourage patients to feel comfortable and disclose more information. Participants built rapport with Ellie at the same level they did with a human therapist. However, when the participants believed that Ellie was controlled by a human rather than being automated, this rapport decreased and the users’ fear of disclosing information increased [53]. The study confirmed the value of the verbal cues and the content of the message rather than the non-verbal cues in building the user-agent relationship which agreed with other studies such as [54].

Other researchers focused on endowing the IVAs with therapeutic knowledge following counselling strategies such as motivational interviewing [50, 54, 55]. There is evidence that the content of the delivered message could be the more important determinant in fostering health behaviour change than the way the IVA interacts with the user (i.e. empathy) [56, 57].

Providing recommendations by a professional clinician/system is not enough for a patient to adhere. The recommendations should be explained clearly with a sense of personalisation— how and why they are relevant to the patient [58]. Thus, an explainable virtual advisor (XVA) should have the ability to explain why a recommendation is given to the user. To date, the role of XVAs in health is not properly investigated as the agents provide explanation in the form of education and guidance to users with less sense of personalisation. An exception is the work [20] and [59] who utilised reason explanation to design explainable agent to educate children with diabetes.

An example of IVAs providing explanations in the form of education and guidance is the study by [38]. With 29 participants, they reported that patients were more likely to sign the consent form to participate in a medical study after they received an explanation of the consent by the IVA rather than a human, particularly those with low health literacy. Participants reported their comfort to discuss, disclose, and to ask the agent to repeat its explanation more than when they interact with a human therapist. Similar results have been reported by [60] with 149 patients who interacted with a virtual discharge nurse to receive instructions and explanation on the discharge procedure and what to do after.

### Explainable agents

Explainable artificial intelligence (XAI) has gained importance with the advancement in automated and persuasive systems. In XAI, explanation can be of two types: data-driven explanation or goal-directed explanation [25]. The data-driven explanation is the interpretation of the output/decision of the machine learning models while the goal-directed explanation, also called explainable agency, is the justification of the agent actions according to its mental state and reasoning process. The majority of work in XAI has been done in the area of data-driven explanation [61]. Little work, but rapidly increasing, has been done in the area of explainable agency/explainable agents (XAs) [25].

People perceive IVAs as social entities and they respond to them socially as they do with other humans [62]. They expect these IVAs to have mental states that derive their behaviour and, thus, they expect agents to be able to explain their behaviours [15]. Hence, it is best to build XAs that can mimic the ways humans explain their behaviour to others which is commonly done by referring to their mental state [11, 19. Found such explanations have been well received by users in terms of understandability.

BDI agents are built to mimic the human cognitive reasoning process using beliefs, desires and intentions [14]. With these elements, an agent derives its actions, and consequently, explains them. The beliefs are the context or the knowledge of the agent about its environment; the goals are the objectives the agents can achieve through possible stored plans in its memory, and the intentions are the plans the agent is currently committed to perform. A BDI agent triggers an action based on its beliefs and/or goals which could be represented using a goal hierarchy tree (GHT) such as the GHT in [63]. In any GHT, the agent’s main-goal is placed at the root of the tree and it can be achieved through one or more sub-goals (the branches of the tree) that could be achieved in a sequential or hierarchical order. The leaves of the tree represent the agent’s actions. For the agent to perform an action, some conditions must be attained. These conditions are the agent’s beliefs and all the beliefs above the action have to be true and, consequently, the goals/sub-goals above are achievable.

[19] tested the usefulness of four different patterns of explanation according to GHT using: the goal one-level above the action, the goal two-levels above the action, the belief(s) above the action, and the next/previous goal and action following the current action depending on the place of the current action on the GHT. Twenty non-expert new firefighting trainers evaluated the four types of explanations within a training scenario. Trainers preferred belief-based explanation to explain the agent’s behaviour when only one action or conditional action(s)/goal(s) were adopted. Goal-based explanations were more preferred but not significantly over the belief-based explanations in procedural actions: a sequence of actions/sub-tasks that will be performed by the agent. However, expert firefighters in a similar scenario preferred goal-based explanations [64].

[20] reported a difference in adults’ explanation preferences compared to children. They designed a robot to educate children with Type 1 diabetes about their disease. The robot is also able to detect the child’s mood and to cheer up the child if a bad mood is detected. Using GHT, they utilised the beliefs and goals that are directly above the current actions to design the explanations. With 19 children and 19 parents, they found that both children and adults preferred goal-based explanation over belief-based explanation but the preference of goals over beliefs was significantly greater in the adults’ group.

Besides beliefs and goals [65], investigated the use of valuing which is defined as *“directly indicating the positive or negative affect toward the action or its outcome”*. About 109 participants evaluated five types of explanations that referred to: valuing and belief, abstract valuing, valuing, belief, and belief and goal. Every participant observed one of three behaviours of a robot that first tries to get a cup of coffee from the kitchen or the coffee shop and then explains its action. The participants evaluated the explanation patterns in terms of being believable, acceptable and comprehensive. Participants preferred to receive different a type of explanation for every behaviour; however, valuing only, and valuing and belief based explanations were the most preferred explanations.

### Hypotheses formation

The findings of the above-mentioned studies provide evidence that explanation patterns using goals or beliefs could be perceived differently. However, the introduced XAs with GHT utilised the beliefs and goals of the agent in both the reasoning and explanation. They did not take into account the human user’s beliefs and goals. This use of the agent’s beliefs and goals in designing explanation patterns could be acceptable when the agent’s actions are related to the agent’s environment. However, such explanations may not be perceived as relevant by the user when the agent is a personal assistant or virtual advisor and the actions should be performed by the user, particularly in the domain of health behaviour change as pointed out by [44] who found that people are more likely to be persuaded to change their behaviour when the delivered message forms a link between their own mental state and the recommended behaviour.

Previously, we explored the role of explanation by comparing the influence of a user’s belief-based XVA to an unexplainable VA (i.e. no explanation) [66]. In the context of reducing study-related stress, the XVA was more successful than the unexplainable VA in encouraging health behaviour change for students with different user profiles. For example, after interacting with the XVA, moderately to highly stressed students showed higher intentions to change their behaviours compared to unstressed students. In this study, we are interested in investigating the role of different explanation patterns on health behaviour change.

While explanation facilitates the transference of knowledge, it is critical to select the proper knowledge to transfer [67]. Found that a complete explanation that describes the entire decision process is more important than a simple explanation to increase the users’ understanding and trust in the explanation. However, when an agent explains all of its underlying process, the explanation would include many beliefs and goals which could be irrelevant [17]. Asserted that the explanation should close a small gap of understanding and not be too lengthy. Thus, besides the belief- and goal- based explanation pattern, we are investigating the impact of extending the explanation pattern by providing explanations based on both goal and belief as follows:

#### Hypothesis 1

(**H1**) There is a difference in terms of intention to change a behaviour between users who receive belief-based explanation, goal-based explanation, and belief*&*goal-based explanation.

Considering the importance of the user-agent relationship in adherence [7, 34], we are exploring if the variation in the explanation pattern can build different levels of user-agent relationship:

#### Hypothesis 2

(**H2**) There is a difference in terms of user-agent relationship between users who receive belief-based explanation, goal-based explanation, and belief*&*goal-based explanation.

Further, as mentioned above, behaviour change or adherence in general can be improved through tailoring and personalising the treatment to the user’s profile, cognition and preferences [43, 44, 58]. So, we expect to find a link between the user’s profile and the variation in their intention to change the recommended behaviours.

#### Hypothesis 3

(**H3**) The change in the user’s intention to do a behaviour is associated with the user’s profile^1^.

Another documented main predictor of adherence is the therapist-patient relationship [34]. A positive relationship was found to be associated with significant reduction in stress [68] and increased adherence, satisfaction and quality of life [33]. Therefore, it is of interest to investigate if the change in the intention to do the behaviours is linked to the user-agent relationship after interacting with user-specific XVAs.

#### Hypothesis 4

(**H4**) The change in the user’s intention to do a behaviour is associated with the user-agent relationship.

## Agent architecture

As above-mentioned in the literature review, the use of BDI agents facilitates explaining the agent’s actions using its beliefs and goals. This is because BDI agents use their cognitive mental state in the reasoning process. Hence, to answer the research question, we extend and evaluate the BDI-based cognitive agent architecture FAtiMA (Fearnot AffecTIve Mind Architecture) [69] as described next.

FAtiMA is an agent architecture that allows the agent to logically reason about its actions according to its emotional and cognitive state. Because we are interested in evaluating the impact of explanation only, we have disabled the emotional appraisal component to control the experiment environment. The agent’s emotions could influence the agent-user relationship [41, 50].

As a conversational agent architecture, the agent with FAtiMA communicates with the user through a designed dialogue. The agent perceives the user through multi-choice utterances available for the user to choose from and it responds to the user (agent’s action) by uttering the sentences which is a result of the agent’s reasoning process. Originally, FAtiMA includes the agent’s memory where the agent’s beliefs about its environment and general knowledge are stored. The action selection component takes mainly the agent’s beliefs and logically processes them to adopt new goals and trigger the proper actions. The memory further includes autobiographical memory where the agent can store full episodes of its interactions with the users so it could guide the agent in its future interaction such as recalling the user’s previous goal to encourage a related current behaviour. This unit is useful for multi-session/long-term user-agent interaction. Thus, we disabled the autobiographical unit for now as our current focus is on evaluating a one-session user-agent interaction.

**Fig. 1:**
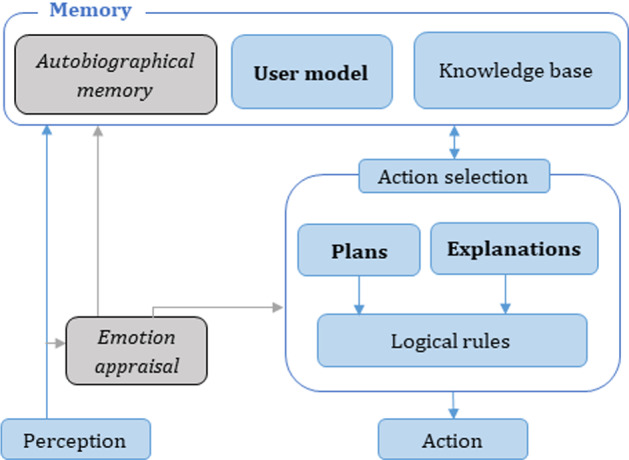
XFAtiMA: the disabled components from original FAtiMA are in italic font and the new components in XFAtiMA are in bold

Towards building an explainable virtual advisor: explainable-FAtiMA (XFAtiMA) that tailors its advice towards the user’s beliefs and/or goals and to refer to them in the explanation process, we added three main units: the user model, plans library, and explanation library. Figure 1 shows the proposed XFAtiMA which includes the components from the original FAtiMA architecture with the disabled components labeled in italic font and the new components specific to XFAtiMA shown in bold. The following subsections describe the new three components in detail.

### The user model

The need for the user model component arises from the need for user-specific reason explanation as discussed in the literature review. Explainable agents can build this mutual understanding when they utilise theory of mind (ToM) in their explanation patterns [70, 71]. The agent, as a virtual advisor, needs to provide explanations by referring to the user’s mental state, particularly beliefs and goals. Previously [72] and [73], added a user model component to FAtiMA-ToM and cultural-FAtiMA, respectively, following the simulation theory of mind. However, in our work, we implement the idea of theory-theory as described in the following paragraph.

There are two main theories of ToM: simulation-theory and theory-theory. In the agent’s memory, the simulation-theory separates the other’s mental states so the agent and every other agent in the environment has a separate mental state. And in every encounter, the agent uses its own reasoning to infer/understand others’ actions. In this sense, the main postulate of this theory is self-similarity [74] such as in multi-agent interaction [71, 75]. However, the assumption of similarity is not applicable in our research context where the agent is a virtual advisor that interacts with human users who have dissimilar minds and reasoning processes. The agent cannot use its reasoning to infer the user’s uncertain behaviour.

Alternatively, in theory-theory, the agent utilises appropriate rules to reason about others’ actions based on their mental states which are stored in the agent’s knowledge base as beliefs. Hence, theory-theory can be implemented with mixed cognitive architectures (human and agent) sharing the same environment. Thus, an agent can have different rules that can be applied according to who is being interacted with: human user or artificial agent. While it is common to implement theory-theory by storing the agent’s beliefs and goals besides the user’s beliefs and goals in the same unit (agent’s knowledge base) (e.g. [71]), we propose to extend the agent’s (*A*) memory to include besides the agent’s beliefs ($B_A$) and goals ($G_A$), in the knowledge base (${KB}_A$), a collection of user models that stores the users’ (*U*) beliefs ($B_U$) and goals ($G_U$) separately. The agent can elicit the users’ beliefs and goals during the interaction and store them in the user model for future use in the reasoning and explanation. The user model can include additional information about the user specific to the context of the interaction such as the medical history in a health scenario or the student’s learning style in an education scenario. However, the current study focuses on evaluating only the user’s beliefs and goals so the user model includes only these two units.

**Fig. 2:**
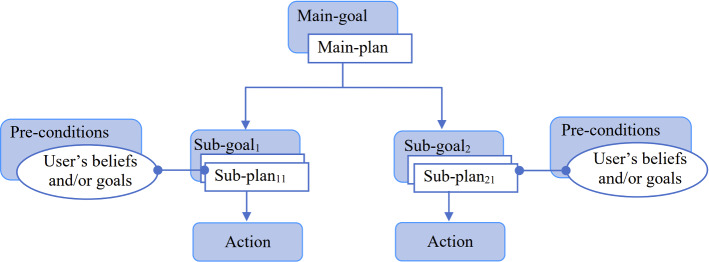
The hierarchical tree of goals and their attached plans in XFAtiMA

### The plans library

**Fig. 3:**
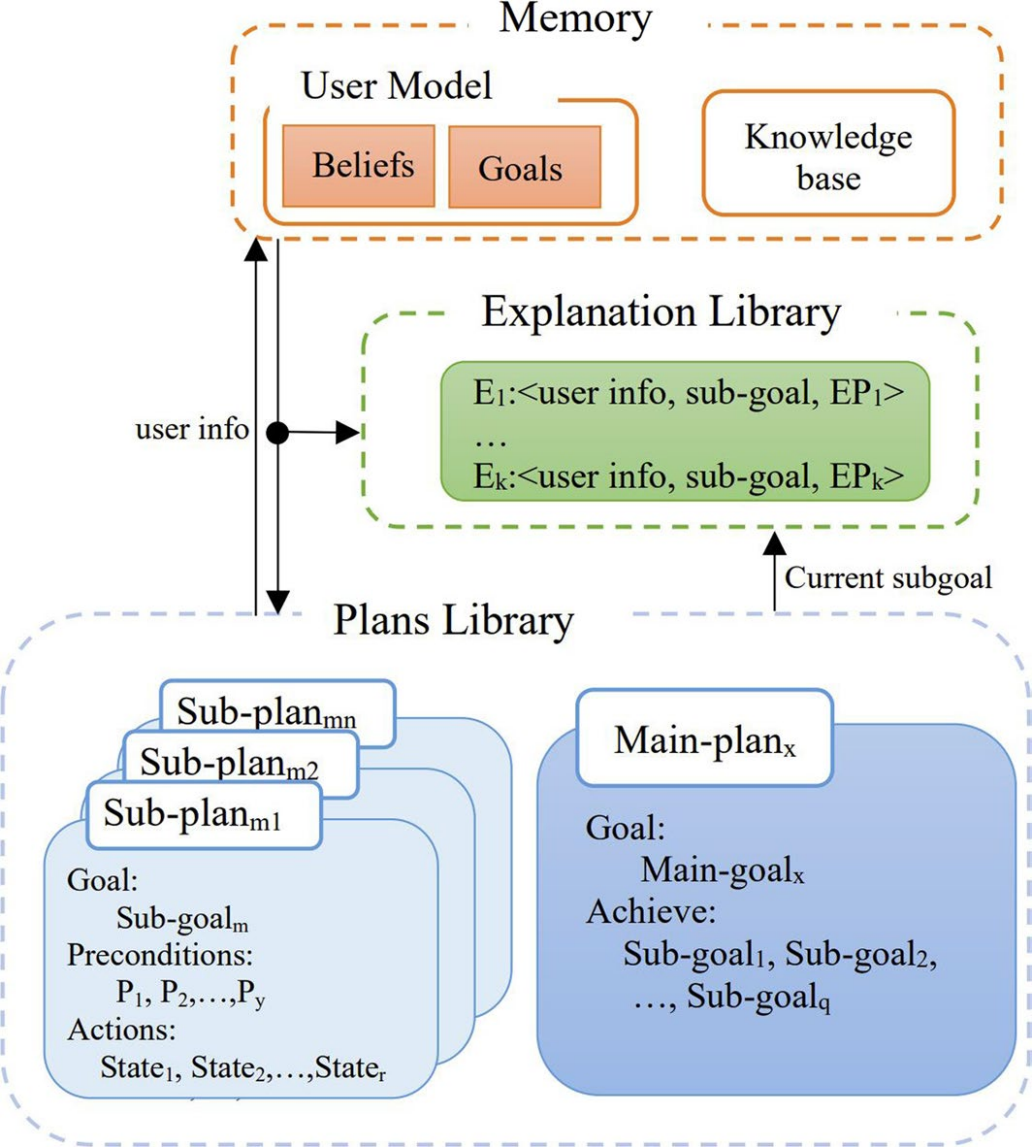
The user model, explanation and plans libraries in XFAtiMA

Inspired by the concept of GHT, as in [19] for example, we designed two types of plans: the main-plan (*MP*) that is designed to achieve a main-goal (*MG*) and the sub-plan (*SP*) to achieve a sub-goal (*SG*). Figure 2 presents a simple GHT and how the plans are attached to the goals in the tree. The root of the tree consists of the main-goal (the agent’s goal of the interaction) and this main-goal is considered attained only when all the sub-goals in the branches of the hierarchy are attained. All these plans are stored in the plan library as illustrated in Fig. 3.

A main-plan is a plan the agent should deliver to the user in one interaction, so it has one main-goal: the interaction goal such as reducing study stress. Hence, a main-plan can be defined as a tuple $<U,MG,SG>$ where: *U* is the user who is the target of the interaction and represented by their user model; *MG* is the main-goal to be achieved through this interaction through a designed main-plan, and *SG* is a list of sub-goals that constitutes the branches of the GHT, as in Fig. 2, under the main-goal, *MG*. For example, the agent may have a main-plan $<X,~weight~loss,~(follow~ a~diet,~exercise)>$ where the agent’s main-goal of interacting with user *X* is to help the user lose weight by recommending a diet and exercise. However, the type of diet and exercise depends on the context of the user *X* and should be tailored to their context (user model). For example, the agent may recommend the user to join a sports team, instead of joining a gym or daily walk, if the user believes the group activity encourages them to do physical activity. Hence, a number of sub-plans (e.g. join a sports team, join a gym and do a daily walk) could be designed to achieve the same sub-goal (recommend exercise) but under different conditions that are related to the user’s context.

Thus, every recommendation is represented as a sub-goal for the agent, and it could be achieved by activating one sub-plan. As presented in Fig. 3, a ${sub-goal}_m$ could be achieved through one of the *n* available sub-plans. A sub-plan is activated only if its preconditions, *Ps*, are satisfied. Hence, as in Fig. 3, a sub-plan is defined as a tuple $<U,SG,P,AC>$ where: *U* is the target/user, *SG* is the sub-goal that could be achieved through this sub-plan, *P* is a list of preconditions that are a set of rules associated with the user information in the user model and *AC* is a sequence of actions to be performed by the agent to attain the associated sub-goal *SG*. As a conversational agent, the *AC* is a dialogue tree represents the turn-taking between the agent and the user where the agent’s action is to utter a statement.

**Fig. 4:**
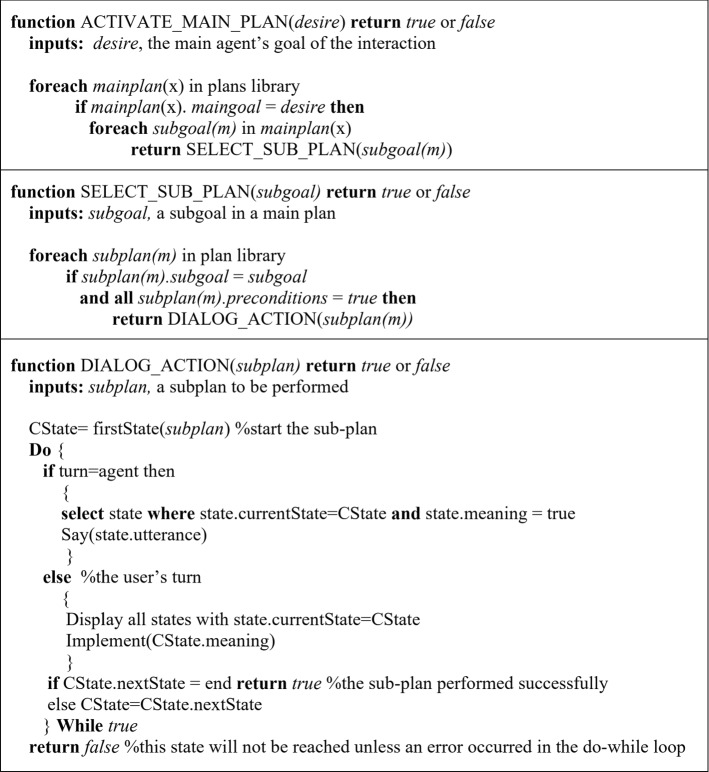
Plans activation algorithm

Figure 4 is a pseudo-code of the algorithm to implement the idea of plans illustrated in Fig. 3, which starts at the beginning of the interaction by taking the agent’s desire (i.e. interaction goal) as an input to the function ACTIVATE_MAIN_PLAN to select the appropriate main-plan from the plans library and implement it by pursuing all the sub-goals listed in the activated main-plan sequentially. The function SELECT_SUB_PLAN takes the agent’s current sub-goal, searches the plans library for a sub-plan that can achieve this sub-goal with all true preconditions. The successful sub-plan is then activated and considered as the agent’s action.

The Actions, *AC*, in a sub-plan is a dialogue tree or states hierarchy. Every state is defined as a tuple $<currentState,~nextState,~meaning,~utterance>$ where: *currentState* and *nextState* are indices to the current and next states in the dialogue tree, respectively, in the dialogue tree; *meaning* could be empty or consist of a list of instructions to be performed. The states in the dialogue tree could be an agent’s state or a user’s state. In an agent’s state, *meaning* could contain preconditions to customise the agent utterances. In a user’s state, *meaning* could contain functions to be performed such as storing the user’s reply as a belief or goal in the user model. Finally, *utterance* is a single statement to be uttered by the agent or to be displayed on the screen as an option for the user to select as a response.

Let’s consider the following example of an agent’s state (*State*1) where the agent can socialise with the user by asking one question that differs according to the day of the week. So the agent has different crafted states for *state*1 to choose from as follows: $<State1,~State2,~day=[Friday],$ “how is your plan going for the weekend?”> and $<State1,~State2,~-~,$ “how is your week going?”>. The state with empty meaning is considered as a default state that is implemented if all other meanings/preconditions return false. After the agent asks the question, the conversation moves in the dialogue tree to *State*2: the user’s turn. Here, all the utterances of the same current state, *State*2, are displayed for the user to select from. For example, to reply to the above agent’s question, the answers could be designed as follows: $<State2,~State3,~belief=[great],$ “It is great”>, $<State2,~State3,~belief=[well],$ “It is going well”>, and $<State2,~State3,~belief=[bad],$ “Not so good”>. Once the user selects their answer, the script in the meaning field, if any, will be implemented: storing the beliefs in the user model. Function DIALOG_ACTION in Fig. 4 presents the implementation of the dialogue tree.

### The explanation library

In the action selection component, an explanation library is added to store all the possible user-specific explanations. The explanation is designed to provide the user with three main aspects: i) the user information: to emphasise the relevancy of the recommendation to the user by stating the user’s belief(s) and/or goal(s), ii) the agent’s active sub-goal: to remind the user about the plan/recommendation provided by the agent, and iii) domain knowledge: extra information relevant to this context explaining how the user could follow the recommendation and how it could help achieving their goal [18, 21].

At this stage of our research, the explanations are pre-crafted to ensure seamless explanations. This library could be replaced by an explanation engine to concatenate the three parts (user info, agent’s sub-goal and domain knowledge) automatically to generate a well-formed explanations. However, in this paper, to focus our research on the value of correctly referring to the user’s beliefs and/or goals and to avoid any syntax mistakes that could be introduced by the explanation engine when it concatenates the three parts of an explanation, we preferred to pre-craft all the potential explanations to be provided to the user. It was possible to take this approach in the this study, pre-crafted explanations, due to the use of constrained input from the user: multi-choice user input.

[11] emphasised the importance of the use of grammatical markers to refer to beliefs: “*I think/believe...*”, goals: “*I want...*”, and to signal subjectivity: “*I/He/She think(s)/want(s)...*”, especially when explaining the behaviours of others. Thus, the an explanation pattern distinguishes between the beliefs and goals by stating the phrase “*you think/find...*” to refer to the user’s beliefs and the phrase “*you want to.,.*” to refer to the user’s goals.

## Methodology

**Fig. 5:**
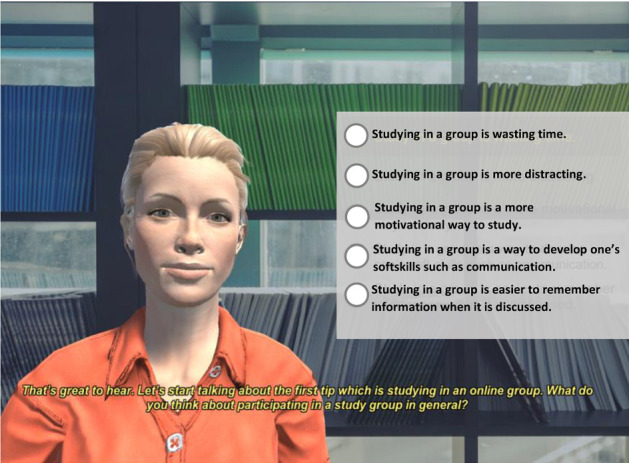
Sarah the XVA for reducing study stress

We designed a virtual advisor (VA), Sarah (Fig. 5), to encourage university students to follow healthy behaviours shown to correlate positively with study stress [76]. We have evaluated Sarah in previous studies to confirm acceptance of interaction with Sarah and that its explanations were found to be sensible and helpful with students in the same context of reducing study stress [77]. Those recommended behaviours were designed carefully by specialists in the university in the Well-being Service Centre and are usually delivered as a pdf or text on the university website.

### Agent dialogue design

The agent starts the conversation by introducing herself, welcoming the user and introducing her goal of the interaction: providing some tips to help manage study stress. The dialogue ends with a farewell conversation. Following the goal tree presented in Fig. 2, the agent’s main-goal in this study is to reduce study stress which could be achieved when a series of sub-goals (i.e. to recommend the behaviours) are completed. The agent recommends three behaviours: participating in a study group, doing regular physical activities and meeting new people. Those three behaviours have been found to be more difficult to change in university students through user-agent interaction [66]. When the agent adopts a sub-goal (to recommend a behaviour), it first checks all the sub-plans designed to achieve this goal. The sub-plan with true pre-conditions is activated and the agent enters into a dynamic conversation with the user to recommend the behaviour. After receiving a recommendation, the user has to ask the agent the *why* question before receiving an explanation.

**Fig. 6:**
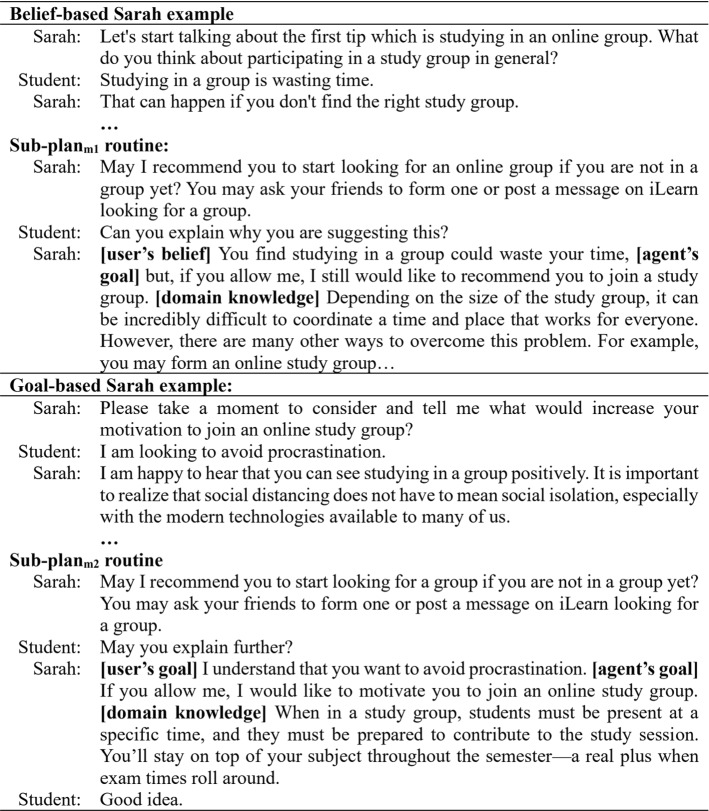
Examples of sub-plans of belief-based and goal-based explainable Sarah including the sub-plans’ routines mentioned in Fig. 2

Figure 6 presents two examples of the conversation with belief-based Sarah and goal-based Sarah. The examples include two sub-plans: ${sub-plan}_{m1}$ and ${sub-plan}_{m2}$ which are segments of state-based dialogue. To elicit the user’s beliefs and/goals, the user is given a list of possible options to select from as described in Sect. 3. As an example, Fig. 5 presents the list of beliefs for the user when Sarah asks about their beliefs about studying in a group. The user’s selection and the rest of the conversation is presented in the belief-based Sarah example in Fig. 6. The ${sub-plan}_{m1}$ is implemented to attain the agent’s ${sub-goal}_m$ (to recommend study in a group) based on the user’s belief (study in a group is wasting time). Appendix A presents a conversation between a student and belief&goal-based Sarah. The conversation is annotated to guide the different segments between brackets such as opening, farewell, small talk, explanations, and the parts of the explanations. The student’s selection from the available list is presented in bold.

### Study design

We designed one XVA (Sarah) with three types of settings: belief-based explanation, goal-based explanation, and belief&goal-based explanation. In the three settings, the agent chats with the user to elicit the user’s beliefs and goals, recommends the same recommendations in a similar order, but it uses different explanation patterns according to the enabled setting. The XVA was designed using the Unity3D game engine and integrated with FAtiMA. The study was approved by the university ethics committee and the participants have been recruited through the university channel where participation was completely optional. The study was listed on the university portal among several other studies for the students to participate in. The students were granted course credit upon completing the study. The study was announced as an online study where the students were able to finish it anytime and anywhere.

Before the interaction, the students received a consent form, and series of questionnaires covering demographics (age, gender, culture), study (achievement aim, if having exam in the following two weeks, course and year of study), personality, propensity to trust and behaviour intention. In the behaviour intention questionnaire, the participants have been asked to rate their intention to do the three activities on 5-point Likert scales (from never to always). Before interacting with the XVA, the students were asked to indicate their emotional feeling towards their studies (stress level) on a scale 0: extremely relaxed to 10: extremely stressed. The scale is designed following the subjective units of discomfort scales (SUD) [78]. Participants are then asked to interact with the assigned XVA. After the interaction, they were asked again to score their study stress level and complete the behaviour intention questionnaire. Moreover, they completed the trust and WA questionnaires.

Although there are several theories to describe personality, the big 5 factors model is a widely used and well regarded personality model [79]. The model is comprised of five factors: extraversion, agreeableness, conscientiousness, openness to new experiences and emotional stability. We used the brief questionnaire developed by [80], called ten-item personality inventory (TIPI), comprising 10 items to measure the five traits using 7-point Likert scales from strongly disagree to strongly agree.

To measure the agent-user relationship, we utilised two questionnaires: trust and WA questionnaires. The WA inventory [81] is a common measurement of the therapist agent-user relationship; however, the built alliance could be a result of a user tendency to trust others in general (trait-like alliance) or of the therapy process (state-like alliance) [82]. Therefore, we included the trust questionnaire which is adapted from [83] to measure the propensity to trust others in general besides trust and trustworthiness sources: ability, benevolence and integrity. The WA questionnaire is the short form of working alliance inventory [81] that measures three elements of WA: task, goal and bond. We also asked the participants to rate their liking for the XVA using one question: “I like the agent”. The trust items were measured using 5-point Likert scales: from strongly disagree to strongly agree. The items in the WA questionnaire and the liking the XVA question were measured on 5-point Likert scale from seldom to always. Further, we provided the participants with the option “Not applicable” next to the scales to choose when they think the question is not applicable to the situation because human-human measures are not always perceived as appropriate for measuring human-agent interactions or relationships [84].

To see whether the explanations had a lasting effect, three weeks after completing the study, participants were sent an email invitation to complete a short follow-up survey containing the same behaviour intention questionnaire for the three behaviours and asking if they actually performed any of the behaviours. The surveys have been designed using Qualtrics and the data has been analysed using SPSS. Due to the use of ordinal data (Likert scales) in the questionnaires and the number of the participants in every group, we opted to use the non-parametric tests in analysing the data [85]. For ease of presentation and explanation of the results, in the tables, we present means and standard deviations.

## Results

### Participants

In total, 91 university students (66 female, 24 male, and 1 other) participated voluntarily in the study and were assigned randomly to one of the experiment groups: belief group ($N=33$, age: $mean=26.00,~median=19.00,~std=12.22$), goal group ($N=34$, age: $mean=24.85,~ median=19.00,~std=11.80$), or belief&goal group ($N=24$, age: $mean=27.67, ~median=28.5, ~std=8.94$). Participants were first year students. In Australia, students do not need to enter university after completing high school but may choose to start tertiary studies at a later time; those students commencing at age 21 and above are considered mature age students [86]. About 53.8% of the participants were under 21-years-old and 46.2% were mature age students (21-years-old and above). Although we randomly allocated participants to each experimental group, the belief&goal group had a higher proportion of mature age students (63%) compared to the belief group (39%) and the goal group (41%) groups.

Participants were from different cultural backgrounds, mainly: 29.7% Oceania, 16.5% Northern-Western Europe, and 16.5% South-East Asian. There were no significant between-group differences in terms of participants’ age ($\chi ^2(4)=1.846$ at $p=.764$), or culture ($\chi ^2(16)=9.977$ at $p=.868$). Table 1 reports the personality test results for the participants in each group. There was no statistically significant between-group difference in terms of personality.

The responses for some variables were significantly different than the normal distribution. We also had some concerns regarding sample size and the use of 5 point Likert scales [87]. Thus, we chose to be conservative and use non-parametric tests.

**Table 1:** Participants distributions among the three groups and their personality stats (E: Extraversion, A: Agreeableness, C: Conscientiousness, O: Openness to experiences, E: Emotional Stability)

Setting	Belief		Goal		Belief&Goal	
	*mean*	*std*	*mean*	*std*	*mean*	*std*
E	4.05	1.66	3.57	1.43	3.71	1.30
A	4.68	0.99	4.91	0.83	4.79	1.18
C	4.79	1.19	4.99	1.46	4.98	1.20
O	4.92	1.02	4.90	1.15	5.00	0.82
ES	3.74	1.49	3.84	1.40	4.02	1.34

### Study stress

Table 2 reports the average scores and standard deviations of the participants’ stress before and after interacting with the XVAs on a scale of 0 to 10. Wilcoxon signed ranks (SR) test revealed that participants showed statistically significant reduction in their study-related stress after interacting with the XVAs in the three groups. The Kruskal-Wallis test reported no statistically significant difference in stress between the three groups before interaction, indicating a fair distribution of the participants among groups. Further, the test reported no significant between-group differences in terms of stress change.

**Table 2:** Study stress before and after comparison

Group	Before interaction		After interaction		Wilcoxon SR test	
	*mean*	*std*	*mean*	*std*	*Z*	*p*
Belief	6.52	2.108	5.21	2.132	-3.543	$\varvec{<.001}$
Goal	5.74	2.416	4.21	2.293	-3.534	$\varvec{<.001}$
Belief&goal	5.58	2.020	4.50	2.187	-2.913	$\varvec{<.01}$

### Behaviour change intentions

Table 3 presents the statistics of the participants’ intentions to do the three behaviours before interacting with the assigned XVA and immediately after the interaction. The analysis reveals significant greater intentions to do the three behaviours after interacting with belief-based XVA and goal-based XVA compared to their intentions before the interaction. Participants who interacted with belief&goal-based XVA showed significant change in their intentions to do physical activity and to meet new people but not to join a study group.

**Table 3:** Behaviour change intentions statistics immediately after interacting with the XVAs

Activity	Before interaction		After interaction		Wilcoxon SR test	
	*mean*	*std*	*mean*	*std*	*Z*	*p*
*Belief-based explanation group*						
Study in a group	2.18	0.92	2.70	0.95	− 3.532	$\varvec{<.001}$
Do physical activity	3.03	1.26	3.48	1.12	− 2.879	**.004**
Meet new people	2.55	1.06	2.97	0.98	− 3.300	**.001**
*Goal-based explanation group*						
Study in a group	2.24	0.89	2.62	0.78	− 2.427	**.015**
Do physical activity	3.03	1.09	3.38	0.95	− 3.207	** .001**
Meet new people	2.56	0.79	3.00	0.92	− 3.095	**.002**
*Belief&goal-based explanation group*						
Study in a group	2.29	0.86	2.46	0.98	− 1.633	.102
Do physical activity	3.25	1.07	3.71	1.12	− 2.598	**.009**
Meet new people	2.54	0.66	3.13	0.68	− 2.841	**.005**

In [66], we found that the intention to change different behaviours varies under different factors. Thus, we stratified the analysis considering relevant factors from the user profile: age, gender, personality, achievement aim (high: distinction and high distinction vs. low: credit and pass), having exam (yes/no), and study stress level.

The Kruskal Wallis test was used to investigate whether the differences in the intention changes between the three experimental groups for each behaviour followed similar patterns for different ages, we separated participants into two age groups: mature age (21 years or older) and under 21-years-old. For mature-age students there was no statistically significant difference at $p<.05$ between the three experiment groups in their intention changes with the three behaviours. For participants under 21 years old, the Kruskal Wallis test reported a between groups significant difference only in the intention change to join a study group ($H=10.244,~df=2,~p<.01$). Mann-Whitney test reported significant differences in the intention change to join a study group between belief and belief&goal groups ($Z=-3.021~at~p=.003$), and between goal and belief&goal groups ($Z=-2.627~at~p=.005$) where participants in both the belief group and goal group showed higher intentions to change than those in the belief&goal group.

For the study stress factor, the stress level was moderately correlated with the change in intention to join a study group in the belief group only (Spearman’s $\rho =.441~at~p=.010$). No further association was found between the changes in the intentions and other factors: gender, personality, achievement aim, and having an exam.

### Behaviour change intentions followup

About 50% (46) of the participants completed the follow-up survey: 19 out of the 33 participants in the belief group (age: $mean=28.26, median=19.00, STD=13.92$), 13 out of the 34 participants in the goal group (age: $mean=27.38, median=21.00, STD=15.74$), and 14 out of 24 in the belief&goal group (age: $mean=27.79, median=30.00, STD=8.85$). The followup group can be seen as representative of the main group who completed part1 of the study (91 participants). Analysis reported no significant differences between the two groups in terms of age ($\chi ^2(26)=12.80$ at $p=.98$), or culture ($\chi ^2(8)=.821$ at $p=.99$). Further, Mann-Whitney reported no significant between-group differences in terms of gender, personality, before the interaction stress level and intentions to change the three behaviours.

Table 4 reports the behaviour change intentions of the 46 participants to do the three recommended behaviours before interacting with the assigned XVA, immediately after the interaction and 3 weeks later. The table further reports the significance of the changes in the intentions to do the behaviours at both points of time after the interaction considering the intentions before the interaction as a baseline using Wilcoxon SR test.

Further, Wilcoxon SR test reported no significant differences between the participants intentions to do the behaviour immediately after the interaction and after 3 weeks later. As mentioned later in discussion, despite their intentions, participants did not carry out the behaviours as recommended by the XVA due to the uncertainty with evolving COVID regulations.

**Table 4:** Behaviour change intentions statistics after 3 weeks of interacting with the XVAs

Activity	Before interaction		After interaction		After 3 weeks	
	Mean	std	Mean	std	Mean	std
*Belief-based explanation group*						
Study in a group	2.11	0.99	2.74**	1.05	2.42$\dagger$	0.90
Do physical activity	3.11	1.41	3.63*	1.12	3.79*	0.976
Meet new people	2.47	1.12	2.89*	1.05	2.84	1.02
*Goal-based explanation group*						
Study in a group	2.15	1.07	2.85*	0.80	2.54	1.05
Do physical activity	2.54	0.98	2.92*	0.76	3.00$\dagger$	1.16
Meet new people	2.69	0.75	2.77	0.83	2.62	0.77
*Belief&goal-based explanation group*						
Study in a group	2.36	0.84	2.50	1.09	2.29	1.07
Do physical activity	2.86	1.10	3.43*	1.22	3.00	0.96
Meet new people	2.71	0.47	3.14$\dagger$	0.66	2.93	0.73

** Significant intention change compared to the baseline at $p<.01$

* Significant intention change compared to the baseline at $p<.05$

$\dagger$ Borderline intention change compared to the baseline at $p=.05$

### User-agent relationship

The participants in the three groups reported average propensity to trust others in general: $mean=3.08$ with $std=.378$ for the belief group, $mean=3.12$ with $std=.445$ for the goal group, and $mean=3.18$ with $std=.344$ for the belief&goal group with no between-group significant difference, which confirms the fair distribution of the participants among the groups. The reliability of the trust and working alliance questionnaires were high with Cronbach’s $\alpha =.914$ and .960, respectively.

**Fig. 7:**
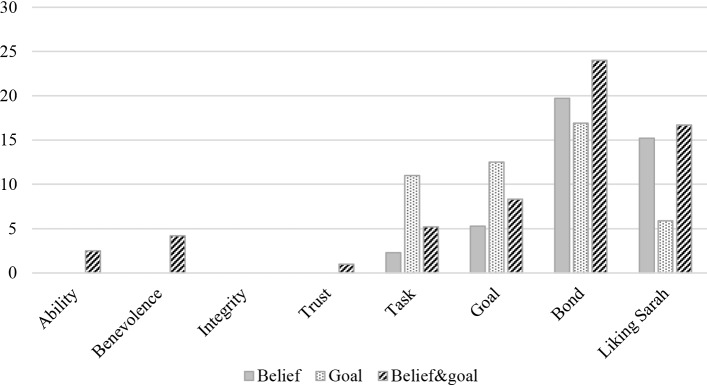
Trust, WA, and liking the agents statistics. The bars presents how many times the option “not applicable” is reported for every construct

Figure 7 reports the results of analysing the total number of times (in percentages) the participants responded to any item of the specified construct as not applicable (NA). Thus, the reported means of the constructs presented in Table 5 are calculated as the average of only the valid responses on the 5-point Likert scales. Table 5 reports the means and standard deviations of the trustworthiness, trust, working alliance, and liking the agent constructs. Kruskal-Wallis test reported no statistically significant differences between the three groups in terms of any of the listed constructs at $p<.05$. Trust was moderately to strongly correlated with ability (Spearman’s $\rho =.656~at~p<.001$), benevolence (Spearman’s $\rho =.461~at~p<.001$), and integrity (Spearman’s $\rho =.547~at~p<.001$).

**Table 5:** Trust, WA, and liking the agents statistics measured on the 5-point Likert scales

Construct	Belief-based		Goal-based		Belief&goal-based	
	Mean	std	Mean	std	Mean	std
*Trust and trustworthiness*						
Ability	3.45	0.667	3.59	0.846	3.46	0.671
Benevolence	3.12	0.919	3.36	1.009	3.30	0.765
Integrity	4.00	0.718	3.99	0.702	4.02	0.634
Trust	2.95	0.863	3.06	0.857	2.94	0.618
*WA*						
Task	2.67	0.936	2.93	1.239	2.80	0.918
Goal	2.45	0.96	2.90	1.195	2.59	1.096
Bond	2.62	1.176	2.92	1.213	2.69	1.162
**Liking the XVA**	2.68	1.124	3.03	1.307	3.00	1.124

### Behaviour change intentions prediction

To study if the user profile and the user-agent relationship, separately, are associated with the intention change as stated in **H3** and **H4**, binary logistic regression was run to explore the factors that can explain the variance in the intention to change the three recommended behaviours. The models’ outcomes (the intentions changes) were coded as 0 for negative and no change in the intentions to do the behaviours, and as 1 for positive changes. Two levels of binary logistic regression models were performed to explore if the source of the variation in the intention change were related to the users’ profiles only or also to the relationship built with the XVA.

In the first level, the factors in the users’ profiles were used as predictors including: age, gender, personality, stress level, study achievement aim, having exam, and the intention to do the behaviour before the interaction with the XVA. We grouped all the participants’ change in the intentions from the three groups for analysis. Explanation pattern type was also listed as a potential predictor in the regression. If the explanation pattern has a significant contribution to the variation in the intention change, it would appear in the list of significant predictors of the models.

The models were statistically significant in predicting the intentions to do the three behaviours. For joining a study group (${\chi }^2(5)=16.820,~at~p=.005$, Nagelkerke $R^2=.230$ (i.e. the explained variation in the dependent variable (intention change) based on the model$=23\%$)). And for doing a daily physical activity (${\chi }^2(3)=14.319,~at~p=.003$, Nagelkerke $R^2=.230$). Finally, for meeting new people (${\chi }^2(3)=25.653,~at~p=<.001$, Nagelkerke $R^2=.339$). Table 6 presents the significant source of variations in the intention changes to do the three behaviours from the user profile according to the three regression models. The constant predicts the variation in the intention change if all the other predictors in the regression model are zero.

In the Second level, the user-agent relationship factors (liking the XVA, trustworthiness, trust, and WA scales) were included as predictors in the regression models. The models were also statistically significant in predicting the intentions to: join a study group (${\chi }^2(5)=26.660,~at~p<.001$, Nagelkerke $R^2=.401$ with 76.9% classification accuracy), do a daily physical activity (${\chi }^2(5)=28.094,~at~p<.001$, Nagelkerke $R^2=.380$ with 72.7% classification accuracy) and meeting new people (${\chi }^2(4)=32.598,~at~p=<.001$, Nagelkerke $R^2=.415$ with 70.5% classification accuracy). Table 7 presents the details of the second level regression models.

**Table 6:** The binary logistic regression models with user’s profile factors only as predictors. Degree of freedom (*df*)=1. SE stands for standard error, and initial intention is the intention to do the behaviour before interacting with the XVA

Predictor	B	SE	Wald	*p*	*E*(*B*)	$(95\%~CI)$
*Behaviour: join a study group*						
Initial intention	−.679	.308	4.998	** .025**	.507	(.274–.919)
Stress level	.321	.136	6.186	**.013**	1.379	(1.069–1.779)
Constant	−3.442	1.864	3.40	.065	.032	–
*Behaviour: do a physical activity*						
Initial intention	−0.783	.297	12.671	$\varvec{<.001}$	.457	(.262–.795)
Agreeableness	.564	.268	4.088	**.043**	1.758	(1.009–3.065)
Constant	-3.411	1.933	3.118	.077	.033	-
*Behaviour: meet new people*						
Initial intention	−1.386	.390	12.248	$\varvec{<.001}$	.250	(.119–.548)
openness to experience	.0.690	.282	5.974	**.015**	1.993	(1.146–3.466)
Having exam(yes)	−1.039	.523	3.939	**.047**	.354	(.127–.987)
Constant	.186	1.269	.021	.884	1.204	-

**Table 7:** The binary logistic regression models with user’s profile and user-agent relationship scales as predictors. Degree of freedom (*df*)=1. SE stands for standard error and initial intention is the intention to do the behaviour before interacting with the XVA

Predictor	B	SE	Wald	*p*	*E*(*B*)	$(95\%~CI)$
*Behaviour: join a study group*						
Initial intention	−.882	.354	6.210	**.013**	.414	(.207–.828)
Age	.045	.026	2.920	.087	1.046	(.993–1.102)
Task	.907	.277	10.708	**.001**	2.477	(1.439–4.264)
Constant	−3.378	1.593	4.495	**.034**	.034	-
*Behaviour: do a physical activity*						
Initial intention	−1.003	.304	10.848	**.001**	.367	(.202–.666)
Agreeableness	.557	.294	3.582	.058	1.746	(.980–3.108)
Openness to experiences	.501	.301	2.770	.096	1.650	(.915–2.975)
Integrity	1.390	.659	4.448	**.035**	4.015	(1.103–14.612)
Trust	.923	.424	4.749	**.029**	2.517	(1.097–5.774)
Constant	−5.443	2.597	4.393	**.036**	.004	-
*Behaviour: meet new people*						
Initial intention	−1.545	.402	14.778	$\varvec{<.001}$	.213	(.097–.469)
Openness to experiences	.670	.297	5.103	**.024**	1.954	(1.093–3.495)
Having exam (yes)	1.411	.590	5.719	**.017**	4.101	(1.290–13.040)
Trust	.952	.381	6.243	**.012**	2.591	(1.228–5.467)
Constant	−3.500	1.742	4.036	**.045**	.030	-

## Discussion

The main research goal of this study is to explore the value of building an XVA that refers to the users’ cognition and to define some factors that link to the desired outcome (behaviour change intention). These factors can help in building an XVA that tailors its advice and explains *why* this particular advice is given by citing the user’s beliefs and goals to increase the sense of personalisation and engagement towards increasing the adherence to the given advice. The influence of the use of belief-based explanation vs goal-based explanation or a combination of both was measured in terms of the desired system outcome: behaviour change intentions and user-agent relationship.

The XVA, designed using the proposed XFatima, delivered three tips to the participating students to reduce their study-related stress. The results showed that students felt less stressed after interacting with the three versions of the XVA (statistically significantly—see Table 2) which indicates that students found the XVAs’ advice relevant and helpful to them in study planning and reducing their stress. Students’ comments at the end of the study confirm this conclusion such as *“It was very interesting to see the advice and responses to my own personal encounters with studying.”*, *“This experiment was useful. It is reflecting on where I was in terms of my study schedule and plans. I will be using the tips provided within my daily routines.”*.

In the first subsection below, we discuss findings concerning the influence of the explanation patterns on behaviour change intention and the user-agent relationship. In the second subsection, we discuss prediction of the user’s behaviour intentions based on the users’ profile and user-agent relationship.

### Explanation patterns

#### Explanation patterns and behaviour change intention

Concerning **H1**, we explored whether there were any significant differences between the three groups in their intentions to change behaviours. Participants in the belief group and goal group reported a significant increase in their intentions to do the three behaviours recommended by the assigned XVA. In the belief&goal group, participants reported significant change in their intentions to do two out of the three behaviours. However, there were no between-groups significant differences in their intention changes to do the three behaviours. Hence, there was no evidence of a difference in terms of intention to change a behaviour between users who receive belief-based explanation, goal-based explanation, and belief&goal-based explanation (**H1**).

Further, as an impact of COVID-19, the participants in the followup survey reported difficulty to do physical activities during the 3 weeks between the interaction with the XVAs and the followup survey. However, their behaviour change intentions were statistically significantly different between baseline (before interaction) and 3 weeks after the interaction in the belief group ($p =0.016$) and means were slightly higher in the goal and belief&goal groups (but not statistically significant) (Table 4). As presented in Table 4, the averages of the intentions to do the behaviours in the three groups were still greater after 3 weeks compared to before the interaction with the XVAs, except in 2 out of the 9 cases (3 behaviours x 3 settings). The belief group showed the highest lasting intentions to do the 3 behaviours with significant differences after 3 weeks at $p<.05$ for one behaviour and at $p<.10$ for two behaviours. Considering the time of the study, during the pandemic, the challenge of behaviour change became even harder: the normal challenge of behaviour change and the additional challenge of adapting to the evolving situation and rules, which could have negatively impacted the participants’ intentions.

#### Explanation patterns and user-agent relationship

Similarly, the analysis did not find any evidence of differences in terms of user-agent relationship between users who received belief-based explanation, goal-based explanation, and belief&goal-based explanation (**H2**). The user-agent relationship was measured using the trust and WA questionnaires. The results are reported in Table 5. The trustworthiness scales (propensity, ability, benevolence, and integrity) were utilised in the study to capture the source of trust in the XVA. The propensity to trust strangers was not correlated with trust in the XVA which indicates that the participants perceived the XVA as trustworthy (state-like trust) and not because they tend to trust strangers (trait-like trust) [82].

Although the participants had the chance to mark the trustworthiness and trust questions as “not applicable”, they did not take that option (Fig. 7) after interacting with belief-based or goal-based XVA, whereas 0%-4.2% did after interacting with the belief&goal-based XVA. The WA results presented in Fig. 7 show that the participants marked the bond questions most frequently as not applicable, compared to the other constructs, to describe their alliance with the XVA. The participants in the belief&goal group scored the highest percentage of perceiving the bond questions as not applicable (24%) which could be due to the long discussion about their beliefs and goals, followed by participants who discussed beliefs only (19.7%) and then the goal only (16.9%). A similar pattern can be noticed with the participants’ responses to how much they liked the XVA. The additional content could have seemed unnatural or too intrusive. A prior study by [88] found that when the agent interferes with the user’s privacy, it could impose a feeling of discomfort. Thus, future work should incorporate measuring how the users perceive the agent asking them to disclose their beliefs and goals to the agent and if this act influences the treatment outcome.

#### Influence of explanation patterns

So far, there is no significant difference between the use of the three patterns of explanations in terms of behaviour change intention or user-agent relationship. However, in our earlier work [66] comparing the influence of interacting with an XVA vs. unexplainable VA, the stratafied analysis according to factors from user profile revealed interesting results about when explanation can be helpful or not. The stratified analysis in this study found slight between-group differences. The stratified analysis in Sect. 5.3 provided explanation for the low intention to change in the belief&goal group. It showed that the low intention to join a study group in the belief&goal group appears only with under 21-year-old students [16]. Argues that longer explanation can inhibit a user’s intention to change which could be a result of increase in the cognitive load. In this study, the belief&goal-based XVA’s dialogue is longer than the belief-based and goal-based dialogues with about 35% and 29% more words, respectively.

Further, highly stressed students can be motivated to join a study group by receiving belief-based explanation where stress level and intention change in the belief group were positively correlated and were more likely to report less stress level after the interaction ($\rho =.459~at~p=.001$). These results indicate that elements from the user profile can be determinants of which type of explanation a user should receive to motivate their intention change. Thus, we turn our focus in that direction in the following subsection.

### Behaviour change intention prediction

Students could safely cope with study stress by following several social, health and planning behaviours [66]. Different factors play roles in defining which behaviour could be best to be recommended to a student over another. Previously, for instance [24], found that personal attitudes impact the intention to change the dieting behaviour but not exercise while perceived control increases the intention to exercise more than dieting. While we conclude above that there is little preference for one explanation pattern over another to motivate the change in one particular behaviour, the differences in the binary logistic models for the 3 behaviours of interest in this study revealed that the process of motivating a user to follow a particular recommendation depends on different factors from the user profile and user-agent relationship.

#### Behaviour change intention and user profile

Results from binary logistic regression (Tables 6 and 7) showed how factors from the user profile can predict (explain some proportion of variability of) the intentions to change. For example, for students having an upcoming exam, the XVA can recommend the student to meet new people to cope with the study stress.

In the university setting, emotional stability has been found to be a significant predictor of stress vulnerability [89]. As a functional stress coping behaviour, students seek study support from university peers, particularly for first year students as they experience higher stress levels compared to others in the following years [90]. In this study, stress level was significantly negatively correlated with emotional stability (Spearman’s $\rho =-.400~at~p<.001$) and, considering the user profile factors only, stress level was the significant predictor of the change in the intentions to join a study group. For every one-unit increase in the stress level, we expect a 1.379 increase in the log-odds of intention to join a study group, holding all other independent variables constant. This can been seen as a compelling reason to use an XVA, particularly belief-based XVA, that can be available any time to motivate students to join a study group and help them deal with their study stress.

Agreeableness was found as a significant predictor of the change in the intention to do physical activity. Prior meta-analysis reported that agreeableness and openness to experiences are weakly to not correlated with doing physical activity; however, this general conclusion, as reported in the meta-analysis, did not take age into consideration where age was found to mediate the association between the agreeableness and physical activity. The effect of agreeableness was clearer with people under 35 years old. About 76.9% of the participants in this study were under 35 years of age, thus, it is more likely to have agreeableness as a predictor for the intention to do physical activity.

Moreover, it is noteworthy to mention that the study has been conducted during the COVID-19 pandemic (February-June 2020). The advice delivered by the XVAs to do the activities has been adapted to suit the government’s guidelines in place during this special time. The XVAs, for example, recommended walking alone or with a friend considering the social distancing and within the restricted local area only. No regular physical activity such as going to a gym or group activities were recommended. Similarly, the XVAs recommended different strategies to meet/interact with new people virtually over the internet. These COVID-19 specific modifications could explain the variation in the results of this study compared to previous findings. Recent studies on personality and complying with the COVID-19 restrictions reported that higher agreeableness is a main predictor of restriction compliance and open to experiences are highly correlated with behaviour awareness during this pandemic [91, 92]. As a result, agreeableness and openness to experiences are the only personality traits found as predictors for the intention change to do physical activity and to meet new people. In conclusion, Tables 6 and 7 identify the relationship between the user’s profile and the variation in their behaviour change intentions, which supports **H3**.

#### Behaviour change intention and user-agent relationship

The two level binary regression models indicate that the defined factors from the user profile only can significantly explain around 23% of the variance in the intention change to join a study group and to do physical activity, and 34% of the variation in the intention change to meet new people as presented in Sect. 5.6. These variances could be explained further, up to 40%, 38%, and 41%, for the three behaviours (Table 7), respectively, by including factors related to the user-agent relationship.

The user-agent trust was associated with the changes in intentions to do physical activity and to meet new people, as shown in Table 7. Participants who trusted the XVA were about 2.5 times more likely to change their intentions to do physical activity and to meet new people than those who did not build trust in the XVA. The XVA’s integrity was significantly associated with the intention change for doing physical activity with high odds ratio equal to 4.015. This positive relationship built with the agent can explain the persistence of the intention to do the behaviours after 3 weeks of the interaction. A similar pattern of association can be noticed between trust and the intention to meet new people. These findings were drawn from the data of all the participants in the three groups and the setting of the experiment (the pattern of explanation) did not contribute to the prediction of the variation in the intention change. In conclusion, the results reported in Table 7 indicate how the user-agent relationship is associated with the variation in the behaviour change intentions, which supports **H4**.

### Limitations and future work

To support the findings of this study, we are interested in several future directions. This study mainly focused on the influence of stating the user’s mental state in the explanation pattern on behaviour change and user-agent relationship. However, to confirm the value of user-specific reason explanations, it is practical to run another randomised controlled trial to compare the value of referring to the users’ mental state over referring to the agent’s mental state in the context of behaviour change. Second, the between-group results provided some indications that participants showed less intention to change after receiving belief&goal-based explanation. Thus, more investigation could be helpful to study if simpler explanation is preferred over longer ones. Third, it is of interest to measure how users perceive XVAs that discuss their beliefs and goals in terms of privacy and see if that impacts the XVAs efficacy for delivering behaviour change or harms the user-agent relationship.

It is noteworthy to mention that this is a context-specific study. Although these elements/factors from the user profile can only be utilised to build a tailored XVA for the same context, it provides evidence of the need for defining the bases or factors for explanation tailoring in other contexts.

Further, the behaviour change was measured by measuring the intention change towards the behaviour following the theory of reasoned action [26]. A followup survey or longitudinal study that measures the change in the users’ actual behaviour will provide clearer evidence of the intervention. Finally, in this study to ensure that all the participants received the assigned type of explanation, the participants had to ask the XVA the why-question before receiving an explanation. Earlier, we found that in some circumstances, the user prefers not to receive an explanation and providing an explanation may undermine the user-agent relationship [66]. Hence, we are designing a future study where the participant has the freedom to ask for explanation or not. This will allow us to study if this optional explanation could maintain the user-agent relationship.

## Conclusion

Prior studies in explainable agency (e.g. [20, 63]) concluded that the use of beliefs or goals in the explanation should be delivered according to the agent’s action or the user’s profile. However, little is known how those types of explanations are perceived by the user when the action should be performed by the user such as those actions recommended by virtual advisors to encourage behaviour change. To potentially improve the ability of VAs to motivate the user to change their behaviour intentions, we have extended the well-known FAtiMA BDI-based cognitive agent architecture with plans library, explanation library and user model to support user-specific explanations. Specifically in this study, we investigated the influence of referring to the user’s beliefs and goals to explain *why* an action should be taken by the user. Three patterns of explanations have been investigated including user’s beliefs, goals, and combined: beliefs and goals. Although there was no difference between receiving the three explanation patterns on the user’s intention to change a behaviour, receiving a longer explanation that includes beliefs and goals tends to some extent hinder the motivation to change the intention to do some behaviours within specific contexts. The preference to receive belief-based or goal-based explanation over belief&goal-based explanation was linked to the user profile with some behaviours (e.g. younger students were less motivated to join a study group after receiving belief&goal-based explanation).

Our study showed that explainable virtual advisors can encourage change in behaviour intention and build a good user-agent relationship, but evidence concerning the influence of different explanation patterns was inconclusive. Across the groups, participants rated the XVAs almost the same in terms of being trustworthy and they built similar levels of WA with the different XVAs. This relationship can explain up to 40% of the variability in intention to change a behaviour after interacting with an XVA along with some factors from the users’ profiles, therefore it can be used for prediction. This finding aligns with the concept of WA: where adherence can be predicted by the level of therapist-patient relationship. Further, different behaviours were found to be predicted by different factors. Defining these factors is a great advance towards building a tailored XVA that can understand the user’s characteristics and context and, then, tailor its advice and explanation accordingly to build a better relationship with the user and motivate behaviour change. While the experiment does not show clear overall difference in behaviour change between the three types of tailored explanation, except for some specific contexts, given the importance and challenge of changing users intention to change their behaviour, it is encouraging that, in general, making the explanation user-specific is adequate to motivate intention to change.

## Appendix A: Dialogue example of belief&goal-based Sarah

*Please note that the text between square brackets are annotations to describe the following statement or variables. The user’s utterances in bold are the responses selected by the user from the available utterances. *

**Sarah**: **[opening/greeting]**Hey, my name is Sarah. I am very happy to meet you and hope you’ll find our session together worthwhile. Please make yourself comfortable. **[Today=Monday]** How was your weekend?

**Student**:    - It was great.    - It was good.    **- It was not that good.**

**Sarah**: Sorry your weekend was not great.

**Student**: Thanks!

**Sarah**: During this pandemic time, you may be worrying about how to manage your study time. The outbreak may be stressful for you as a student.

**...**

**Sarah**: **[small talk]**Now let us talk about physical activity which helps in reducing study stress. Now, as more of us are living and working or studying in the same space, it is even more important to take active breaks to move, stretch and make the time to focus on our physical and mental well-being. Do you do any daily exercise?

**Student**:    - No.    -Yes.

**- Currently, I am not exercising but I am planning to start doing that soon.**

**Sarah**: That is a good plan. Please tell me what you think about doing daily exercise?

**Student**: **[user’s belief]** Exercising is:    - boring.

- beneficial but I do not have time to exercise for about 30 minutes a day.

**- a trend nowadays where everyone does a daily exercise.**

- only for people who have a health problem.

- good for everyone’s body and mind as well.

**Sarah**:Right, lots more people are exercising and going to the gym nowadays. What do you want to achieve through exercising?

**Student**: **[user’s goal]** I want to:    - build muscles.    - lose weight.    - have fun.

**- maintain my mental health.**    - maintain healthy body.

- I am not motivated to do it.

**Sarah**: Exercise benefits every part of your body, including your mind. I recommend you maintain a daily exercise habit. While you should abide by the mandates from your state and local authorities, going outside almost everyone can still do. I found doing an online workout at the same time or even just chatting about exercising with my friends motivating to keep exercising as a habit at this time.

**Student**: Please explain.

**Sarah**: **[user’s belief]** You feel that doing daily exercise is a trend these days. **[agent’s sub-goal]** I agree with you, but I would like to clarify why people tend to act like that. **[domain knowledge]** Every day brings with it a new scientific report on the benefits of exercise. It does not only help in losing weight, but regular exercise helps prevent or manage many health problems and concerns like stroke, type 2 diabetes and anxiety. Exercising also causes the body to make chemicals that can help a person to feel good and sleep better.

**Student**: I would like to hear more explanation.

**Sarah**: **[user’s goal]** You want to maintain your mental health through exercise. **[agent’s sub-goal]** I admire your goal and I strongly encourage you to keep your goal always on the top especially during the quarantine. **[domain knowledge]** Exercising causes the body to make chemicals that can help a person to feel good. Exercise can help people sleep better. It can also help some people who have mild depression and low self-esteem. Plus, exercise can give people a real sense of accomplishment and pride at having achieved a certain goal.

**Student**:    **- Sounds dumb.**    - Sounds good.

**Sarah**: It is always up to you to decide. Hope you can find your own way to maintain your health. Quarantine should not prevent us from staying active. You do not need any equipment to stay active. Online workouts, dog walking, jogging, dancing, using the stairs are a few examples of exercising.

**Student**: Right! Let us move on.

**...**

**Sarah**: **[farewell]** Okay, our time is nearly over. My last tip is to remember we are bound to feel stressed and a little overwhelmed during tough study time – it is usually an indicator of our motivation to do well! Looks like mission accomplished. Good luck. It has been nice chatting with you. Thanks for participating in this experiment. If you need some support in the future, I would love to chat with you again. Take care and see you.

## Footnotes

1 We use the word profile to include the user’s personal details (e.g. age, gender and personality) and his/her context (e.g. study aims, upcoming exams, study stress level).
